# Supplementary material for: Dnmt1 mediates epigenetic restriction of invasive traits in clonal crayfish
Source: Nat Commun. 2026 Mar 26;17:2954. doi: 10.1038/s41467-026-71049-z (PMC13031303; doi:10.1038/s41467-026-71049-z)
Supplement: Supplementary file 1 — Supplementary Infomation [file 41467_2026_71049_MOESM1_ESM.pdf]

Supplementary information

Dnmt1 mediates epigenetic restriction of invasive traits in clonal crayfish

J. Jaime Diaz-Larrosa[1], Vitor Carneiro[1], Katharina Hanna[1], Günter Raddatz[1] and Frank Lyko[1,*]

[1]Division of Epigenetics, DKFZ-ZMBH Alliance, German Cancer Research Center, 69120, Heidelberg, Germany.

*Correspondence:

Frank Lyko: f.lyko@dkfz.de

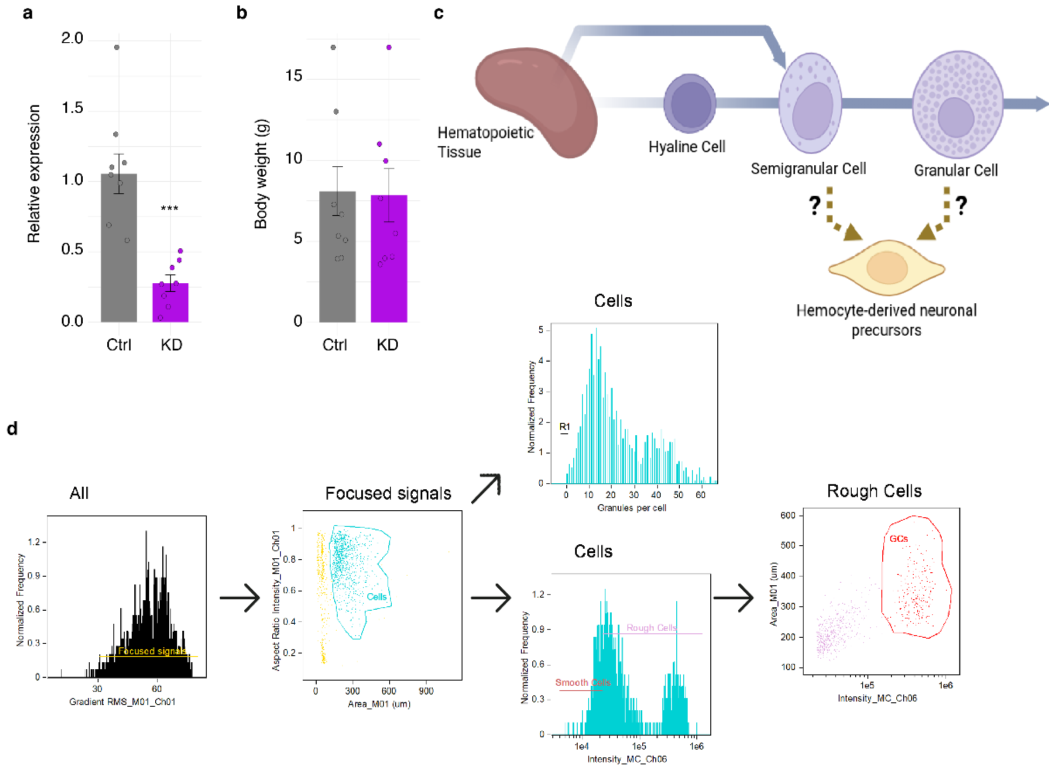

Supplementary Fig. S1. Details of behavioral experiments and image cytometry gating strategy. a, *Dnmt1* transcript levels measured post-behavioral assays confirmed successful knockdown in *Dnmt1* KD animals (n=8, purple) compared to controls (n=9, gray). Statistical analysis was performed using an unpaired two-sided t-test (p-val=0.0002) and 95% confidence intervals. Bars represent mean values ± standard error, and each dot represents a biological replicate. **b**, Animal weights measured prior to dsRNA injection showed no significant difference between control (n=9) and KD groups (n=8), ensuring weight-matched comparisons. Statistical analysis was performed using an unpaired two-sided t-test (p-val=0.91) and 95% confidence intervals. Bars represent mean values ± standard error, and each dot represents a biological replicate. **c**, Schematic model summarizing hemocyte development in marbled crayfish hemolymph created in BioRender. Diaz, J. (https://BioRender.com/u4q6ehu) is licensed under CC BY 4.0. **d**, Image cytometry gating strategy for the identification of the three main immune cell types. Starting from all measured cells, focused signals were isolated, and single cells were identified. HCs were defined as cells with 0-1 granules (side scatter R1). Based on side scatter intensity, focused signals were further divided into smooth and rough cells; rough cells were separated by size to isolate GCs. SGCs were defined as cells neither categorized as HCs nor GCs. Control = Ctrl; Dnmt1-Knockdown = KD; ***p<0.001. Source data are provided as a Source Data file.

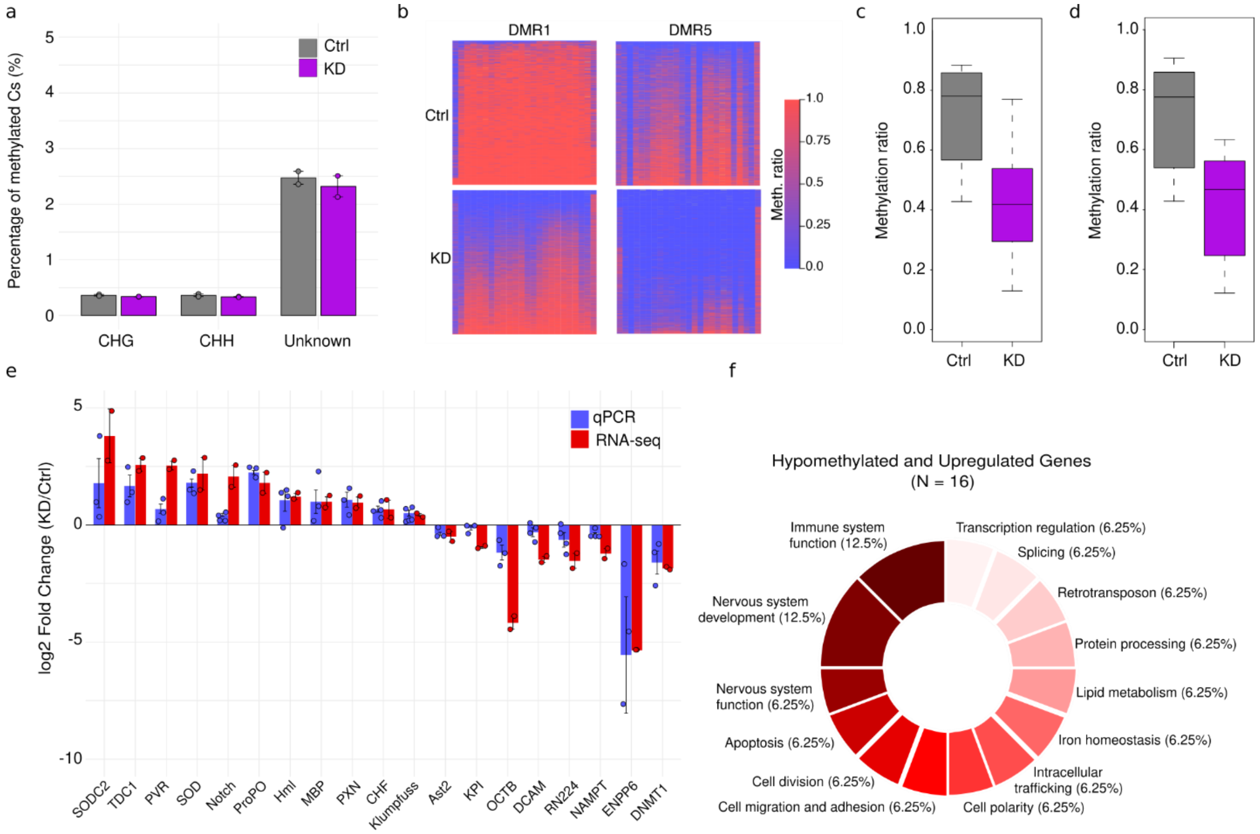

Supplementary Fig. S2. DNA methylation context, *Dnmt1* knockdown validation, and RNA-seq confirmation. a, Relative abundance of 5-methylcytosines (5mC) in different sequence contexts (CHG, CHH, unknown: CN, CHN) shows that non-CpG methylation represents only a small fraction of total 5mCs and is unaffected by *Dnmt1* knockdown, in contrast to the significant CpG methylation changes shown in Fig. 4a. Bars represent mean values ± standard error and each dot a biological replicate. Unpaired two-sided t-tests showed no statistical significance (Ctrl vs KD; CHG: p-val=0.24; CHH: p-val=0.36; Unknown p-val=0.56) and 95% confidence intervals. **b**, Representative heatmaps of two differentially methylated regions (DMR1 and DMR5) in Ctrl and KD groups, used for rapid assessment and validation of *Dnmt1* knockdown efficiency. **c**, Quantification of global methylation levels in hemocytes 28 dpi and **d**, six months post-injection (mpi) reveals a consistent ~40% reduction in methylation in KD samples compared to controls. Each experiment used n=3 biological replicates per group; statistical analysis was performed using unpaired two-sided t-tests giving for **c**: p-val=0.017; and **d**: p-val=0.0003 and 95% confidence intervals. All box plots within this figure indicate the median (horizontal line), interquartile range (25th-75th percentile), and whiskers representing the minimum and maximum values within 1.5X the interquartile range. **e**, qPCR validation of RNA-seq results. Fifteen

randomly selected DEGs ($p<0.05$), three non-DEGs ($p>0.05$) showing an upregulation trend, and *Dnmt1* were analyzed. Log2 fold changes between KD and control hemocytes (28 dpi) showed trends consistent with the RNA-seq data, supporting the reliability of the transcriptomic analysis. qPCR group sizes: n=3, 4 or 5 biological replicates; Bars represent mean values ± standard errors. **f,** Functional annotation of hypomethylated and upregulated genes revealed a broad distribution of biological functions without notable enrichment patterns. Abbreviations: *SODC2*, superoxide dismutase [Cu-Zn] 2; *TDC1*, tyrosine decarboxylase; *PVR*, PDGF/VEGF receptor; *SOD*, superoxide dismutase; *ProPo*, prophenoloxidase; *Hml*, hemolectin; *MBP*, mannose-binding protein; *PXN*, peroxinectin; *CHF*, crustacean hematopoietic factor; *AST2*, astakine 2; *KPI*, Kazal protease inhibitor; *OCTB*, octopamine receptor beta-1R; *DCAM*, S-adenosylmethionine decarboxylase proenzyme; *RN224*, RING finger protein 224; *NAMPT*, nicotinamide phosphoribosyltransferase; *ENPP6*, glycerophosphocholine cholinephosphodiesterase ENPP6; *Dnmt1*, DNA methyltransferase 1. Source data are provided as a Source Data file.

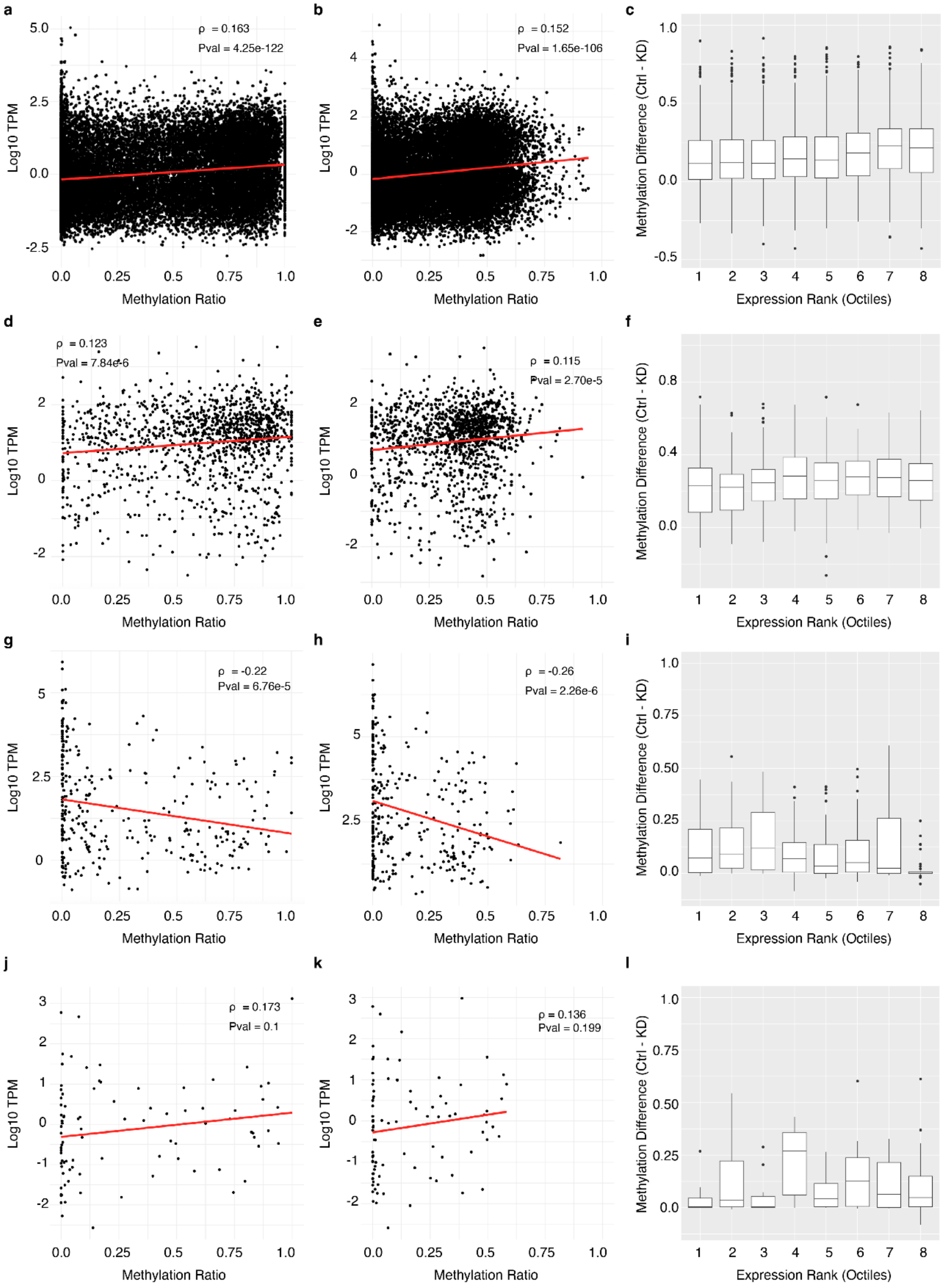

Supplementary Fig. S3. WGBS & RNA-Seq integration shows gene class-specific correlations between gene body DNA methylation and gene expression. Integration of WGBS and RNA-seq data, using averaged methylation ratios and TPMs across biological

replicates, reveals distinct correlation patterns: **a-c,** Across *all genes* (n=20503), gene body methylation and gene expression show a significant positive correlation in both **a**, control and **b**, knockdown samples. **c,** When gene expression is divided into octiles (1=lowest, 8=highest), against the loss of methylation (meth ratio Ctrl – KD) the highest-expressed genes show the largest methylation loss upon knockdown, indicating a positive link between methylation loss and expression level. **d-e,** Housekeeping genes (n=1322) follow the general trend but display a bell-shaped distribution when grouped by **f**, octiles, with moderately expressed genes losing the most methylation, a pattern reported in previous studies. **g-i,** Immune genes (n=323) show a strikingly different behavior, with a significant negative correlation between methylation and expression in both **g,** control and **h**, KD, and methylation loss concentrated in **I**, the lower-expression bins. **j-l,** Neuronal genes show no clear correlation, suggesting that methylation-expression relationships may be tissue-specific. Every row represents a gene class, left panels are always control and middle panels KD samples. Pearson´s product-moment correlation tests were performed in R, reporting both rho and p-values on the plots. All box plots within this figure indicate the median (horizontal line), interquartile range (25th-75th percentile), and whiskers representing the minimum and maximum values within 1.5X the interquartile range.

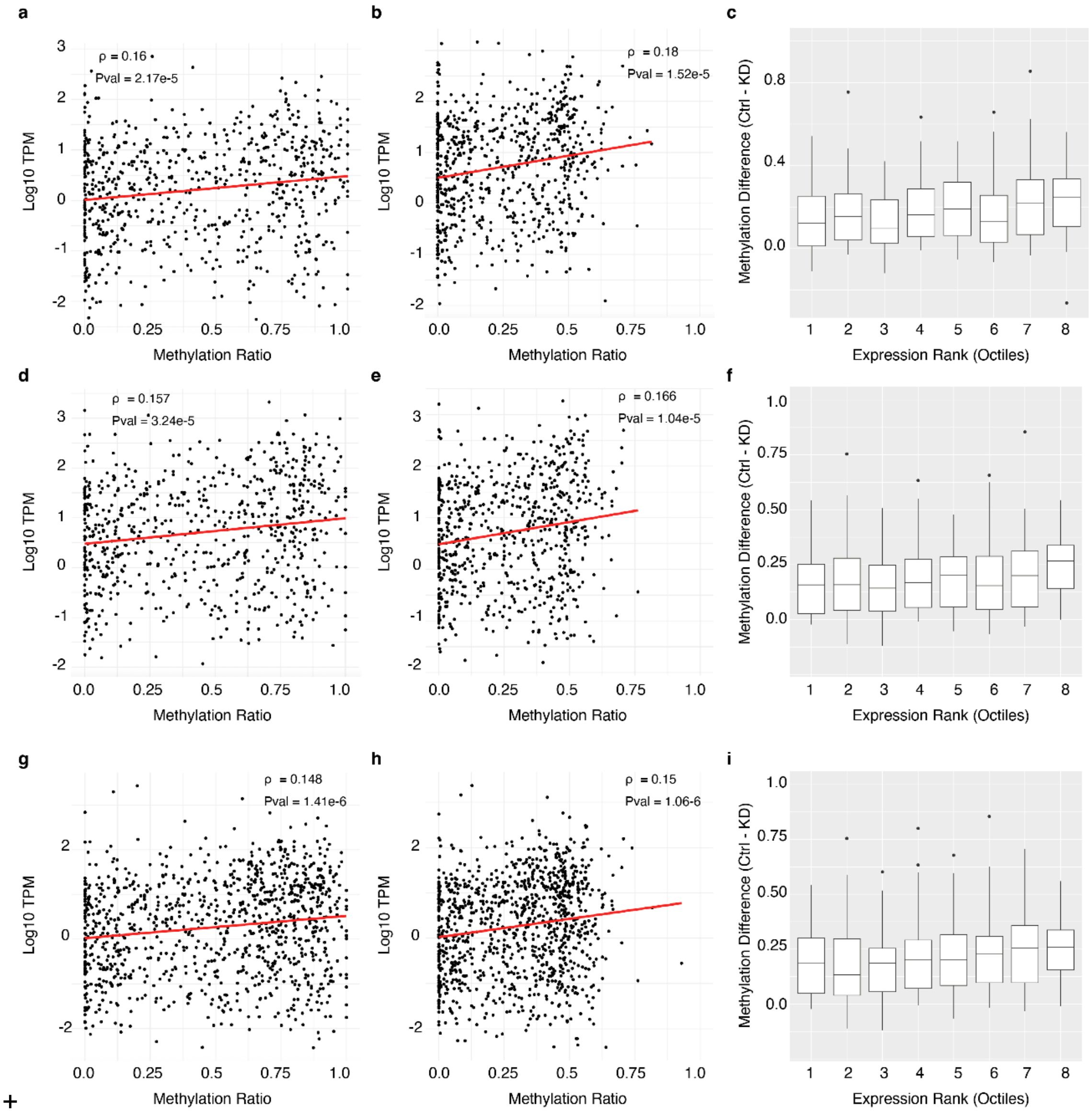

+

Supplementary Fig. S4. WGBS & RNA-Seq integration of other gene classes. a-c, Transcription factors, **d-f,** hematopoietic genes, and **g-i,** meiotic genes all show a positive correlation between methylation loss and expression, especially prominent in the highest-expression octiles, though some lower-expression bins also show substantial methylation loss. Every row represents a gene class, left panels are always control and middle panels KD samples. Pearson´s product-moment correlation tests were performed in R, reporting both rho and p-values on the plots. All box plots within this figure indicate the median (horizontal line), interquartile range (25th-75th percentile), and whiskers representing the minimum and maximum values within 1.5X the interquartile range.

Supplementary Tab. S1. Single-cell RNA-seq details.

Sample	Seurat Object ID	Group	Cells before QC	Cells after QC	Median genes/cell	Median UMI/cell
**Ctrl1**	dsGFP1	Control	8078	5318	114	244
**Ctrl2**	dsGFP2	Control	1201	1196	491	1135
**Ctrl3**	dsGFP3	Control	650	622	330	720
**KD1**	dsD1	Dnmt1 Knockdown	894	881	548	1246
**KD2**	dsD2	Dnmt1 Knockdown	999	635	111	196
**KD3**	dsD3	Dnmt1 Knockdown	3008	2869	175	584

Supplementary Tab. S2. Whole Genome Bisulfite Sequencing (WGBS) details.

Sample	Group	Raw reads	Depth	Depth after filtering <10X Cs	Mapping efficiency (%)	Conversion ratio (%)
**Ctrl1**	Control	440,958,485	10.4 X	20.8 X	37.8	99.75
**Ctrl2**	Control	221,623,053	6.6 X	16.4 X	44.8	99.75
**KD1**	Dnmt1 knockdown	442,198,371	9.8 X	20.4 X	35.3	99.78
**KD2**	Dnmt1 knockdown	189,161,366	5.6 X	15.2 X	45.1	99.77

Supplementary Tab. S3. Bulk RNA-seq details.

Sample	Comments	Raw reads	Q30 (%)	Mapped reads	Assigned reads
**Ctrl1**	Control	128,197,599	98.11	99,682,544	24,544,189
**Ctrl2**	Control	145,459,006	98.11	115,150,632	30,313,705
**KD1**	Dnmt1 knockdown	143,109,174	98.12	115,695,245	31,115,241
**KD2**	Dnmt1 knockdown	137,260,101	98.12	108,976,724	28,226,744

Supplementary Tab. S4. MNase-seq details.

Sample	Comments	Number of reads	Mapping efficiency (%)	Depth
**Ctrl1**	Control	215,442,954	93.42	5.16 X
**Ctrl2**	Control	224,839,317	93.47	5.34 X
**Ctrl3**	Control	238,783,060	92.93	5.66 X
**Ctrl4**	Control	328,033,441	95.21	7.9 X
**KD1**	Dnmt1 knockdown	259,977,259	93.38	6.2 X
**KD2**	Dnmt1 knockdown	246,807,606	94.03	5.94 X
**KD3**	Dnmt1 knockdown	237,997,578	94.67	5.74 X
**KD4**	Dnmt1 knockdown	361,241,752	95.22	8.65 X
**KD5**	Dnmt1 knockdown	290,871,684	95.61	7.02 X
**KD6**	Dnmt1 knockdown	306,508,449	95.62	7.38 X

Supplementary Tab. S5. Overview of replicates used per experiment/method.

Method/experiment	Control biological replicates (n)	Dnmt1-KD biological replicates (n)	Figure
**Cold effect on *Dnmt1* expression**	6	6	1a
**Cold effect on *Dnmt3* expression**	3	3	1b
**Cold effect on *Tet* expression**	3	3	1c
**Biofloc effect on *Dnmt1* expression**	3	3	1d
***Dnmt1*-KD effect on *Dnmt1* expression (several tissues)**	3	3	1e-l
**Behavioural analysis**	9	8*	2a-f & S1a-b
**Image Cytometry**	6	6	2h-j
**scRNA-seq**	3	3	3
**WGBS**	2	2	4a-c & S2a
**Targeted bisulfite sequencing 28 dpi (WGBS validation)**	3	3	S2b-c
**Targeted bisulfite sequencing 6 mpi (WGBS validation)**	3	3	S2d
**RNA-seq**	2	2	4d-e
**qPCR (RNA-seq validation)**	3-5†	3-5†	S2e
**MNase-seq**	4	6	5f-l

*One KD individual was excluded from behavioral analysis due to lack of locomotion (see Methods). †Number of biological replicates varied slightly between genes analyzed.

Supplementary Tab. S6. Primer sequences for qPCR.

Primer	Sequence (5´- 3´)
TBP_qRT_forward	GCTCGGATTGTACAGAAGTTGG
TBP_qRT_reverse	GGCTGTGAGTGAGGACAAGG
Dnmt1_qRT_forward	GCCAGCGTTTACCTCAGAAG
Dnmt1_qRT_reverse	ACCCGCATGGTTAGTTTGAG
SODC2_qRT_forward	AGTGGCAGGCTGGAAATCTA
SODC2_qRT_reverse	CTTCTCGTGCACATGGAAGC
TDC1_qRT_forward	AGGTTCGAGTCCCATGTGAG
TDC1_qRT_reverse	ACCATGTGAAGCTTCCCTGA
PVR_qRT_forward	CCTCAGCACGTATTTCCAGC
PVR_qRT_reverse	GGAAACAGTAGTCCTCGCCT
SOD_qRT_forward	TACGGTGTATGGGCTGACTC
SOD_qRT_reverse	CTTGAAGGGGTTGAAGTGGC
Notch_qRT_forward	AGATGCGAGACAGACACCAA
Notch_qRT_reverse	TTACTCCCATGTACGCCTCC
ProPo_qRT_forward	ATCGAGGGGTCGTGTTTCAA
ProPo_qRT_reverse	CGTCGATAACTTTGGCCAGG
Hml_qRT_forward	AGATTCTGCTCTCGTCTCCG
Hml_qRT_reverse	CGGCGAAGGTTTCACAAGAA
MBP_qRT_forward	AGCCTGTGTTGACTCCTACC
MBP_qRT_reverse	GCTGCCTATGACGTACTCCT
PXN_qRT_forward	CTTCCCAATCCGCGTCAAAT
PXN_qRT_reverse	AGGGAAGGGAACATGAGCAA
CHF_qRT_forward	ATGTCCCAAGGTGCAGTGTA
CHF_qRT_reverse	GGTTCCAAATGCCTCCACAT
Klumpfuss_qRT_forward	CAGACCTGGTGCGCAATATC
Klumpfuss_qRT_reverse	GAGGTGTTTGAGCCGAGTTG
Ast2_qRT_forward	GCTGCAATTTCTCCACTCGT
Ast2_qRT_reverse	TACGAGGTGGAAGAAGCGAG
KPI_qRT_forward	GGTGGGTTATGTGGACGGTA
KPI_qRT_reverse	TCTGTACAGGCTCCTTCGTG
NAMPT_qRT_forward	AATGTTGTCTTCGGCAGTGG
NAMPT_qRT_reverse	CGTCTTACCGAGGTCAGTGA
DCAM_qRT_forward	TCAGCTGCAAATTCCAAGACA
DCAM_qRT_reverse	TTGCGGATGAGAGTCACACT
RN224_qRT_forward	TGAGGAGGCTGAGACTGTTG
RN224_qRT_reverse	CCAGTGCAGCATCCATTGTT
OCTB_qRT_forward	CGACTCCACTCCACTGCTAA
OCTB_qRT_reverse	CGGAGGCATTGAAGGTCATG
ENPP6_qRT_forward	GCACGATGACATCCCCAATC
ENPP6_qRT_reverse	TGGAGCCCATGATGAAGTGT

Supplementary Tab. S7. Primer sequences to amplify dsRNAs.

Lowercase letters in primer sequences correspond to the T7 promoter required for in vitro transcription, while uppercase letters represent gene-specific sequences used for dsRNA synthesis.

Primer	Sequence (5´- 3´)
dsDnmt1_forward	taatacgactcactatagggATCAGAGGGCAGCTCCAGTA
dsDnmt1_reverse	taatacgactcactatagggCGTAAACAGGGAGAGCTTCG
dsGFP_forward	taatacgactcactatagggGACGTAAACGGCCACAAGTT
dsGFP_reverse	taatacgactcactatagggGGGGTGTTCTGCTGGTAGTG

Supplementary Tab. S8. Primer sequences for MiSeq.

Lowercase letters indicate adapter sequences required for MiSeq library preparation while uppercase letters correspond to target-specific primer sequences.

Primer	Sequence (5´- 3´)
DMR1_MiSeq_forward	tcgtcggcagcgtcagatgtgtataagagacagGTTTGTTTTGGTTTTTTYGGG
DMR1_MiSeq_reverse	gtctcgtgggctcggagatgtgtataagagacagCCRACCCTAACCTACCTAATA
DMR2_MiSeq_forward	tcgtcggcagcgtcagatgtgtataagagacagAYGTATGGATTTTTGGTTATGG
DMR2_MiSeq_reverse	gtctcgtgggctcggagatgtgtataagagacagAAAACTAAACCRACCACCAAA
DMR3_MiSeq_forward	tcgtcggcagcgtcagatgtgtataagagacagTTGAAGTAGGGGAAAGAGGT
DMR3_MiSeq_reverse	gtctcgtgggctcggagatgtgtataagagacagACTCTCCTAACTATTCACRTAC
DMR4_MiSeq_forward	tcgtcggcagcgtcagatgtgtataagagacagGGGGGATTTYGAAGGAATTA
DMR4_MiSeq_reverse	gtctcgtgggctcggagatgtgtataagagacagTCCCTCTCAAATTCRAACTTA
DMR5_MiSeq_forward	tcgtcggcagcgtcagatgtgtataagagacagGGTTAYGTTGTTGGTTTTTTAG
DMR5_MiSeq_reverse	gtctcgtgggctcggagatgtgtataagagacagACRTAATAAAAAACACCACCA
DMR8_MiSeq_forward	tcgtcggcagcgtcagatgtgtataagagacagGGTTTGTTTGGTGAGTATTGG
DMR8_MiSeq_reverse	gtctcgtgggctcggagatgtgtataagagacagACTTCRCCATACTAATCTACC
DMR9_MiSeq_forward	tcgtcggcagcgtcagatgtgtataagagacagATTTGTTGAAAGAGGYGGA
DMR9_MiSeq_reverse	gtctcgtgggctcggagatgtgtataagagacagCTCCCAAATATAAACCTCTTCR
DMR10_MiSeq_forward	tcgtcggcagcgtcagatgtgtataagagacagGTTGGTTTYGTAGTTAGTGGT
DMR10_MiSeq_reverse	gtctcgtgggctcggagatgtgtataagagacagAAATACCCAACRATCAACCTC

Supplementary note 1

List of recurrently used abbreviations and corresponding definitions.

Abbreviation	Definition
**BF**	Bright field
**bp**	Base pairs
**CA**	Climbing attempts
**CPHs**	Circulating prohemocytes
**Ctrl**	Control
**DEGs**	Differentially expressed genes
**DMRs**	Differentially methylated regions
**Dnmt1 / DNMT1**	DNA (cytosine-5)-methyltransferase 1
**dpi**	Days post-injection
**dsDnmt1**	Double-stranded RNA targeting *Dnmt1*
**dsGFP**	Double-stranded RNA targeting *GFP*
**dsRNA**	Double-stranded RNA
**GCs**	Granular cells
**HDNPs**	Hemocyte-derived neuronal precursors
**HCs**	Hyaline cells
**HPT**	Hematopoietic tissue
**KD**	Knockdown
**MNase-seq**	Micrococcal nuclease sequencing
**mpi**	Months post-injection
**PCA**	Principal component analysis
**qPCR**	Quantitative PCR
**RAS**	Recirculating aquaculture system
**RNA-seq**	RNA sequencing
**RT**	Room temperature
**SAD**	Stop average duration
**scRNA-seq**	Single-cell RNA sequencing
**SGCs**	Semigranular cells
**SSC**	Side scatter
**TFs**	Transcription factors
**TPM**	Transcripts per million
**TSC**	Total space changes
**TTL**	Total time in light
**TTI**	Total time immobile
**UMAP**	Uniform manifold approximation and projection
**WGBS**	Whole-genome bisulfite sequencing
**5mC**	5-methylcytosine
