# Supplementary material for: Dnmt1 mediates epigenetic restriction of invasive traits in clonal crayfish
Source: Nat Commun. 2026 Mar 26;17:2954. doi: 10.1038/s41467-026-71049-z (PMC13031303; doi:10.1038/s41467-026-71049-z)
Supplement: Supplementary file 2 — Reporting Summary [file 41467_2026_71049_MOESM2_ESM.pdf]

nature portfolio

Corresponding author(s): Prof. Dr. Frank Lyko
Last updated by author(s): Jun 18, 2025

Reporting Summary

Nature Portfolio wishes to improve the reproducibility of the work that we publish. This form provides structure for consistency and transparency in reporting. For further information on Nature Portfolio policies, see our Editorial Policies and the Editorial Policy Checklist.

Statistics

For all statistical analyses, confirm that the following items are present in the figure legend, table legend, main text, or Methods section.

n/a	Confirmed	
☐	☒	The exact sample size (*n*) for each experimental group/condition, given as a discrete number and unit of measurement
☐	☒	A statement on whether measurements were taken from distinct samples or whether the same sample was measured repeatedly
☐	☒	The statistical test(s) used AND whether they are one- or two-sided<br>*Only common tests should be described solely by name; describe more complex techniques in the Methods section.*
☐	☒	A description of all covariates tested
☐	☒	A description of any assumptions or corrections, such as tests of normality and adjustment for multiple comparisons
☐	☒	A full description of the statistical parameters including central tendency (e.g. means) or other basic estimates (e.g. regression coefficient) AND variation (e.g. standard deviation) or associated estimates of uncertainty (e.g. confidence intervals)
☐	☒	For null hypothesis testing, the test statistic (e.g. *F*, *t*, *r*) with confidence intervals, effect sizes, degrees of freedom and *P* value noted<br>*Give P values as exact values whenever suitable.*
☒	☐	For Bayesian analysis, information on the choice of priors and Markov chain Monte Carlo settings
☒	☐	For hierarchical and complex designs, identification of the appropriate level for tests and full reporting of outcomes
☐	☒	Estimates of effect sizes (e.g. Cohen's *d*, Pearson's *r*), indicating how they were calculated

Our web collection on statistics for biologists contains articles on many of the points above.

Software and code

Policy information about availability of computer code

Data collection	Gene expression data using qPCR were collected using LightCycler 480 SW 1.5.1. Image cytometry data were collected using INSPIRE software
Data analysis	Video recording were analysed using BORIS (v.8.27.10). Image cytometry data were analyzed using IDEAS software. scRNA-seq raw reads were processed with the CellRanger v4.0.0, and analyzed using Seurat v4.3.0 and SeuratObject v4.1.3 R packages; Slingshot (v2.6.0) was used to perform pseudotime analysis. WGBS were processed using Bismark (v0.20.0) and Bowtie2 (v2.3.5.1) for alignment and methylation calling. Targeted bisulfite sequencing were anayzed using BisAMP. RNA-seq raw data were mapped using Hisat2 (v2.2.1) and analyzed using DESEq2 (v1.38.3). MNase-Seq raw reads were aligned using bwa (v0.7.15) and transformed into BED using bedtools (v2.24.0). Analysis was performed using NucTools. Plots were generated using different packages: gplots (v3.1.3.1), ggplot2 (v3.4.4), vioplot (v0.4.0) and heatmap.2.

For manuscripts utilizing custom algorithms or software that are central to the research but not yet described in published literature, software must be made available to editors and reviewers. We strongly encourage code deposition in a community repository (e.g. GitHub). See the Nature Portfolio guidelines for submitting code & software for further information.

Data

Policy information about availability of data

All manuscripts must include a data availability statement. This statement should provide the following information, where applicable:

- Accession codes, unique identifiers, or web links for publicly available datasets
- A description of any restrictions on data availability
- For clinical datasets or third party data, please ensure that the statement adheres to our policy

Single-cell RNA sequencing (scRNA-seq) data generated in this study have been deposited in the NCBI Gene Expression Omnibus (GEO) under accession code GSE295870 [https://www.ncbi.nlm.nih.gov/geo/query/acc.cgi?acc=GSE295870]. Whole-genome bisulfite sequencing (WGBS) data generated in this study are available in GEO under accession code GSE295869 [https://www.ncbi.nlm.nih.gov/geo/query/acc.cgi?acc=GSE295869]. Bulk RNA sequencing (RNA-seq) data generated in this study have been deposited in GEO under accession code GSE295871 [https://www.ncbi.nlm.nih.gov/geo/query/acc.cgi?acc=GSE295871]. MNase-seq data generated in this study are available in GEO under accession code GSE295867 [https://www.ncbi.nlm.nih.gov/geo/query/acc.cgi?acc=GSE295867].
Source data are provided with this paper.

Research involving human participants, their data, or biological material

Policy information about studies with human participants or human data. See also policy information about sex, gender (identity/presentation), and sexual orientation and race, ethnicity and racism.

Reporting on sex and gender	No human participants were used in this study
Reporting on race, ethnicity, or other socially relevant groupings	No human participants were used in this study
Population characteristics	No human participants were used in this study
Recruitment	No human participants were used in this study
Ethics oversight	No human participants were used in this study

Note that full information on the approval of the study protocol must also be provided in the manuscript.

Field-specific reporting

Please select the one below that is the best fit for your research. If you are not sure, read the appropriate sections before making your selection.

☒ Life sciences ☐ Behavioural & social sciences ☐ Ecological, evolutionary & environmental sciences

For a reference copy of the document with all sections, see nature.com/documents/nr-reporting-summary-flat.pdf

Life sciences study design

All studies must disclose on these points even when the disclosure is negative.

Sample size	No sample size calculation was performed, but a minimum of three independent biological samples per group and experiment were used to achieve statistical significance. Whenever this was not possible, an orthogonal validation approach was performed, i.e. MiSeq for WGBS and qPCR for RNA-seq
Data exclusions	Video recordings of animals that displayed no locomotion during the 15 minutes of video were discarded
Replication	Gene expression analysis using qPCR was done in at least triplicates (performed once or two independent times). Three independent batches of control and knockdown groups with n=3 each (total n=9 and n=8 respectively) were video recorded for behavioural analysis. Image cytometry experiments were performed in six different samples per treatment group (two independent batches). Single-cell RNA sequencing were performed using three control and three knockdown hemolymph samples (once). Whole-genome bisulfite sequencing and bulk transcriptomics were performed on two control and two knockdown animals (once)<br>micrococcal nuclease sequencing was performed in four control and six knockdown samples (once). All replications were successful and conclusions based on all samples.
Randomization	Animals were allocated into Control or Knockdown group randomly ensuring groups with similar weights and life cycles stages.
Blinding	Video recording and analysis of behavioural data was done blindly until the end of the process.

Reporting for specific materials, systems and methods

We require information from authors about some types of materials, experimental systems and methods used in many studies. Here, indicate whether each material, system or method listed is relevant to your study. If you are not sure if a list item applies to your research, read the appropriate section before selecting a response.

Materials & experimental systems

n/a	Involved in the study
☐	☒ Antibodies
☒	☐ Eukaryotic cell lines
☒	☐ Palaeontology and archaeology
☐	☒ Animals and other organisms
☒	☐ Clinical data
☒	☐ Dual use research of concern
☒	☐ Plants

Methods

n/a	Involved in the study
☒	☐ ChIP-seq
☐	☒ Flow cytometry
☒	☐ MRI-based neuroimaging

Antibodies

Antibodies used	anti-histone H3 antibody (Abcam, Cat. No.: ab1791; Lot number: 1068998-2) - 4 µg of antibody were used per 5 µg of chromatin.
Validation	This antibody is a rabbit polyclonal antibody used to detect Histone H3. It was used to increase the yield and purity of nucleosome-bound chromatin for MNase-Seq as done for Mallm et al. 2019 doi: 10.15252/msb.20188339. This antibody has been referenced in 5687 publications and in at least 9 different species, some of them non-model organisms. https://www.abcam.com/en-us/products/primary-antibodies/histone-h3-antibody-nuclear-marker-and-chip-grade-ab1791

Animals and other research organisms

Policy information about studies involving animals; ARRIVE guidelines recommended for reporting animal research, and Sex and Gender in Research

Laboratory animals	Procambarus virginalis (marbled crayfish), female, 1-3 years old, of similar weight and life cylce were used in this study. No strains or substrains exist from this species. But all animals were born and raised in the laboratory. It is a clonal parthenogenetic species so no genetic background differences exist.
Wild animals	This study did not involve wild animals
Reporting on sex	P. virginalis is an all-female clonal population that reproduces via parthenogenesis. All used individuals were female.
Field-collected samples	This study did not involve field-collected samples
Ethics oversight	All laboratory experiments were performed by approval of the Zentrum für Präklinische Forschung from the Deutsches Krebsforschungszentrum (DKFZ) animal welfare committee, in compliance with local standards and guideline (Regierungspräsidium Karlsruhe)

Note that full information on the approval of the study protocol must also be provided in the manuscript.

Plants

Seed stocks	No plant species were used in this study
Novel plant genotypes	No plant species were used in this study
Authentication	No plant species were used in this study

Flow Cytometry

Plots

Confirm that:

☒ The axis labels state the marker and fluorochrome used (e.g. CD4-FITC).

☒ The axis scales are clearly visible. Include numbers along axes only for bottom left plot of group (a 'group' is an analysis of identical markers).

☒ All plots are contour plots with outliers or pseudocolor plots.

☒ A numerical value for number of cells or percentage (with statistics) is provided.

Methodology

Sample preparation	Total filtered hemolymph was pelleted and resuspended in 1% dilution of paraformaldehyde (PFA) in water where it was fixed for 20 min at RT.
Instrument	Cytometer ImageStream X MKII from Merck Millipore
Software	Data collection was performed with INSPIRE software and data analysis with IDEAS software
Cell population abundance	Crayfish hemolymph is composed of three main cell types: hyaline cells (0 - 10%), semigrnaular cells (~80%) and granular cells (5-15%)
Gating strategy	Gating strategy was done based on cell size and granularity as differential parameters between crayfish hemocytes. The initially recorded sample population (n=1000) was filtered to keep the focused signals (Gradient RMS M01 Ch01 > 30), and single cells (Area M01 vs Aspect Ratio Intensity M01 Ch01). Cells were then split into smooth and rough cells based on the (Intensity MC Ch06, >1.5e5 associated to rough cells). Using differential size and granularity (Intensity MC Ch06 vs Area Ch01) granular cells were identified as cells with an area > 200 µm2 and a high scatter intensity (> 1e5) and making use of the separation between cell populations. All cells were visually inspected. Spot Count function which allows to count the number of spots, i.e. granules in the side scatter channel (Ch06) was used to identify the hyaline cells as those containing 0 or 1 granules. Those cells that were not classified as either GCs or HCs, were considered SGCs. Signals associated with non-cellular identities, i.e. debris, were discarded.

☒ Tick this box to confirm that a figure exemplifying the gating strategy is provided in the Supplementary Information.
